# Occurrence of Zearalenone and Its Metabolites in the Blood of High-Yielding Dairy Cows at Selected Collection Sites in Various Disease States

**DOI:** 10.3390/toxins13070446

**Published:** 2021-06-28

**Authors:** Wojciech Barański, Magdalena Gajęcka, Łukasz Zielonka, Magdalena Mróz, Ewa Onyszek, Katarzyna E. Przybyłowicz, Arkadiusz Nowicki, Andrzej Babuchowski, Maciej T. Gajęcki

**Affiliations:** 1 Department of Animal Reproduction with Clinic, Faculty of Veterinary Medicine, University of Warmia and Mazury in Olsztyn, Oczapowskiego 13, 10-718 Olsztyn, Poland; wojbar@uwm.edu.pl (W.B.); arkadiusz.nowicki@uwm.edu.pl (A.N.); 2 Department of Veterinary Prevention and Feed Hygiene, Faculty of Veterinary Medicine, University of Warmia and Mazury in Olsztyn, Oczapowskiego 13, 10-718 Olsztyn, Poland; lukaszz@uwm.edu.pl (Ł.Z.); magdzia.mroz@gmail.com (M.M.); gajecki@uwm.edu.pl (M.T.G.); 3 Institute of Dairy Industry Innovation Ltd., Kormoranów 1, 11-700 Mrągowo, Poland; ewa.onyszek@iipm.pl (E.O.); andrzej.babuchowski@iipm.pl (A.B.); 4 Department of Human Nutrition, Faculty of Food Sciences, University of Warmia and Mazury in Olsztyn, Słoneczna 45F, 10-719 Olsztyn, Poland; katarzyna.przybylowicz@uwm.edu.pl

**Keywords:** zearalenone, mastitis, ovarian cysts, pyometra, hepatic portal system, dairy cows

## Abstract

Zearalenone (ZEN) and its metabolites, alpha-zearalenol (α-ZEL) and beta-zearalenol (*β*-ZEL), are ubiquitous in plant materials used as feed components in dairy cattle diets. The aim of this study was to confirm the occurrence of ZEN and its selected metabolites in blood samples collected from different sites in the hepatic portal system (posthepatic–external jugular vein EJV; prehepatic–abdominal subcutaneous vein ASV and median caudal vein MCV) of dairy cows diagnosed with mastitis, ovarian cysts and pyometra. The presence of mycotoxins in the blood plasma was determined with the use of combined separation methods involving immunoaffinity columns, a liquid chromatography system and a mass spectrometry system. The parent compound was detected in all samples collected from diseased cows, whereas α-ZEL and β-ZEL were not identified in any samples, or their concentrations were below the limit of detection (LOD). Zearalenone levels were highest in cows with pyometra, where the percentage share of average ZEN concentrations reached 44%. Blood sampling sites were arranged in the following ascending order based on ZEN concentrations: EJV (10.53 pg/mL, 44.07% of the samples collected from this site), ASV (14.20 pg/mL, 49.59% of the samples) and MCV (26.67 pg/mL, 67.35% of the samples). The results of the study indicate that blood samples for toxicological analyses should be collected from the MCV (prehepatic vessel) of clinically healthy cows and/or cows with subclinical ZEN mycotoxicosis. This sampling site increases the probability of correct diagnosis of subclinical ZEN mycotoxicosis.

## 1. Introduction

Zearalenone (ZEN) is a resorcinic acid lactone produced by fungi of the genus *Fusarium*, which, due to its structural similarity to 17β-estradiol and affinity to estrogen receptors [1], is classified as mycoestrogens. In mammals, ZEN binds to estrogen receptors that display tropism for female reproductive cells. Zearalenone poses a health risk for dairy cows because it is highly stable in contaminated animal feedstuffs and difficult to degrade through heating and other physical treatments [2].

High-yielding dairy cows are highly susceptible to metabolic diseases such as milk fever, ketosis and rumen acidosis, which are frequently accompanied by subclinical and clinical symptoms of udder infection and decreased reproductive performance. Metabolic disorders are most frequently observed in three critical periods [3]: (i) dry period, (ii) parturition, and (iii) first 100 days of lactation. These periods are characterized by an increased risk of udder inflammation and aseptic diffuse inflammation of the laminar corium, which are indicative of compromised innate immunity, decreased acquired immunity to infectious factors [4], and higher susceptibility to undesirable substances, including secondary metabolites of molds such as ZEN and its metabolites.

In dairy cows, susceptibility to mycotoxins is largely determined by the degree to which these substances are eliminated by ruminal microbiota before they are assimilated by the body [5]. Very few mycotoxins are resistant to microbial detoxification in the rumen, and they cause typical symptoms of poisoning with toxic metabolites. Silage and other stored feedstuffs can contain mycotoxins that possess antibacterial properties and modify ruminal microbiota. These compounds can compromise the detoxification capacity of rumen microorganisms. The rumen contents contaminated with mycotoxins may be absorbed in the duodenum, leading to certain mycotoxin concentrations in dairy cows. The clinical symptoms of mycotoxin poisoning are generally non-specific, and they include metabolic and hormonal disorders accompanied by inflammatory states caused by a weakened immune response [4]. During transition periods, cows are particularly susceptible to mycotoxicosis because the presence of molds and/or mycotoxins in cattle diets further deepens the negative energy balance [3].

In dairy cows, the risk of exposure to ZEN can be assessed directly by identifying mycotoxins in the feed matrix [6], or indirectly by analyzing the respective biomarkers in biological fluids and tissues. Feed matrices are widely applied to evaluate the risk [7] resulting from e.g., the presence of ZEN and its metabolites. However, this approach has several limitations. The feed production process and the health status of animals may affect the bioavailability of ZEN and its metabolites in feed and, consequently, increase the risk of exposure in dairy cows. In addition, mycotoxins are not evenly distributed in the feed matrix, and feed intake is difficult to estimate accurately. Since ZEN is transmitted by numerous vectors (green fodder, roughage and concentrated fodder or water), the levels of this mycotoxin and its metabolites can be more reliably quantified in blood samples (indirect analysis).

Our observations show that during clinical activities, field veterinarians need to be aware of some simple information about mycotoxins: (i) mycotoxins are very often found in small amounts in the plant material used for feed production; (ii) not every laboratory has equipment for detecting the presence of very small amounts (these may be values below the sensitivity of the method); (iii) mycotoxin contamination may occur during the vegetation of feed crops, and the mycotoxins are then evenly distributed throughout the plant material; (iv) the feed may become contaminated with mycotoxins during the storage of the final product (concentrated feed) and then the distribution of eg ZEN may be pinpointed in the feed; (v) the accumulation in the macro-organism of low doses of mycotoxins (below the NOAEL value) taken in the feed takes a very long time, making it impossible to trace the mycotoxin vector; and (iv) ZEN is mycoestrogen and its presence in the mammalian body is immediately noted in the form of endocrine system dysfunction and the resulting perturbations in estrogen-dependent tissues.

In cattle, mycotoxicoses are diagnosed based on the presence of clinical symptoms of infertility or hormonal disorders during exposure to high ZEN doses. However, the influence of very low, measurable concentrations of ZEN on the health status of dairy cows has never been investigated. Such mycotoxin concentrations are frequently encountered in plant materials that are used in the production of feed for dairy cattle [6,7,8].

Mycotoxins exert various effects on the health status of animals exposed to their different doses [9,10]. The symptoms and health consequences (toxicological) of exposure to high doses of most mycotoxins have been relatively well researched and described [10]. Prolonged exposure to low monotonic doses of mycotoxins is usually well tolerated by monogastric animals [11], which suggests that these compounds can meet the animals’ life needs, or exert protective [8,12,13,14,15] or therapeutic effects [16]. In ruminants, exposure and digestion processes are similar to those observed in monogastric animals when the rumen contents pass into the intestines. Therefore, the clinical presentation of ZEN mycotoxicosis is also similar [14].

During exposure to low mycotoxin doses, the dose-response relationship has been also undermined by the low dose hypothesis. The above applies particularly to hormonally active chemical compounds [17], including mycoestrogens such as ZEN and its metabolites which disrupt the functioning of the hormonal system, even when ingested in small quantities [18]. This ambiguous dose-response relationship does not justify direct analyses or meta-analyses of the risk (clinical symptoms or the results of laboratory analyses) associated with low dose stimulation and high dose inhibition, which is consistent with the hormesis paradigm [19]. The concept of the lowest identifiable dose which produces counter-intuitive effects is becoming increasingly popular in biomedical sciences [20]. The relevant mechanisms should be investigated to support rational decision-making [8,21]. Such decisions involve the selection of blood sampling sites which are most adequate for assessing the risk of mycotoxin exposure in dairy cows. The main routes of blood inflow and outflow to/from the liver should be examined taking into account the availability of blood vessels and the extent of natural (hepatic) detoxification. Topographic anatomy of anastomoses between the portal venous system (*vena portae*) with the major veins [22], including the external jugular vein (EJV, *v. jugularis externa-*posthepatic), the abdominal subcutaneous vein (ASV, *v. epigastrica cranialis superficialis*-prehepatic) and the median caudal vein (MCV, *v. caudalis mediana*-prehepatic) should be analyzed to confirm the reliability and consistency of the results [4].

The aim of the study was to confirm the occurrence of ZEN and its selected metabolites in the blood of high-yielding dairy cows and selected collection sites (external jugular vein, abdominal subcutaneous vein and median caudal vein) in various disease states (Mastitis, ovarian cysts and Pyometra), in natural conditions.

## 2. Results

### 2.1. Clinical Observations

Clinical signs of ZEN mycotoxicosis were not observed during the experiment (such as reduced growth rate and milk yield, and causes significant economic cost to the dairy industry). However, cows may have been exposed to natural sources of ZEN in plant materials. Cattle diets probably contained very small amounts of the mycotoxin that approximated the minimal anticipated biological effect level (MABEL). Zearalenone transmission vector/vectors could not be identified with full certainty.

### 2.2. Concentrations of Zearalenone in Peripheral Blood

Alpha-ZEL and β-ZEL were not detected in blood samples, or their concentrations were below the limit of detection (LOD).

**Figure 1:**
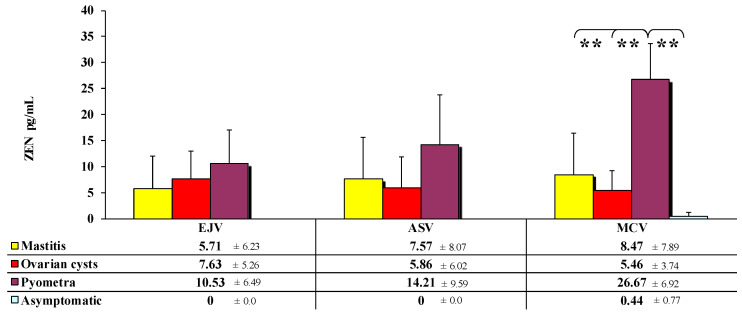
Mean values ($\bar{x}$) and standard deviation (SD) of ZEN concentrations (pg/mL) in peripheral blood sampled from different sites: (1) external jugular vein (EJV, *v. jugularis externa-*posthepatic); (2) abdominal subcutaneous vein (ASV, *v. epigastrica cranialis superficialis*-prehepatic); (3) median caudal vein (MCV, *v. caudalis mediana*-prehepatic) of cows diagnosed with mastitis, ovarian cysts and pyometra, and asymptomatic cows. Limit of detection (LOD) > values below the limit of detection were regarded as equal to 0. Differences were regarded as statistically significant at ** *p* ≤ 0.01.

Highly significant (*p* ≤ 0.01) differences in ZEN concentrations (Figure 1) were observed only in blood samples collected from the MCV. These differences were noted between cows with pyometra compared to blood samples from cows with mastitis (difference of 18.20 pg/mL), cows with ovarian cysts (difference of 21.21 pg/mL) or asymptomatic cows (difference of 26.23 pg/mL). Regardless of the sampling site, ZEN levels were highest in cows displaying clinical symptoms of pyometra. In cows with mastitis and pyometra, ZEN concentrations were lowest in blood sampled from the EJV (5.70 pg/mL and 10.53 pg/mL, respectively) and highest in samples collected from the MCV (8.47 pg/mL and 26.67 pg/mL, respectively. For ovarian cysts, the trend was the opposite between collection sites (from 7.63 pg/mL at EJV to 5.46 pg/mL at MCV).

**Figure 2:**
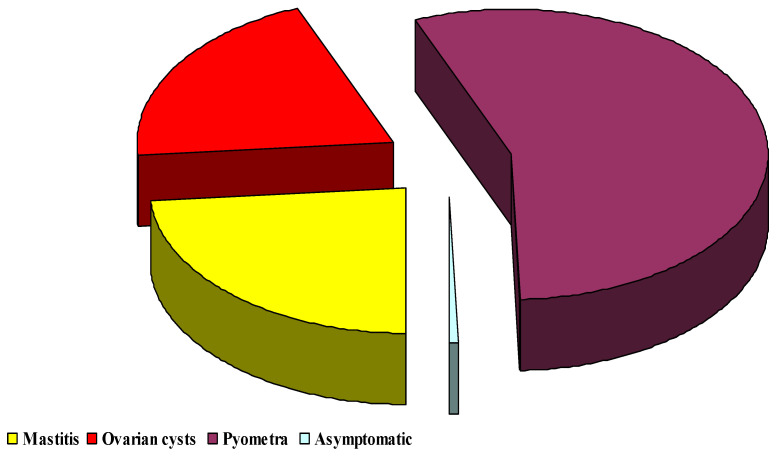
Percentage share of the average ZEN concentrations in blood sampled from cows diagnosed with mastitis, ovarian cysts and pyometra and from asymptomatic cows in the monitored herd.

At the same time, it was found that the mean highest ZEN value (number of samples: the sum of the ZEN concentration values in these samples) was obtained in samples taken from median caudal vein (which accounted for 57%), compared to the other sampling sites (Figure 2).

**Figure 3:**
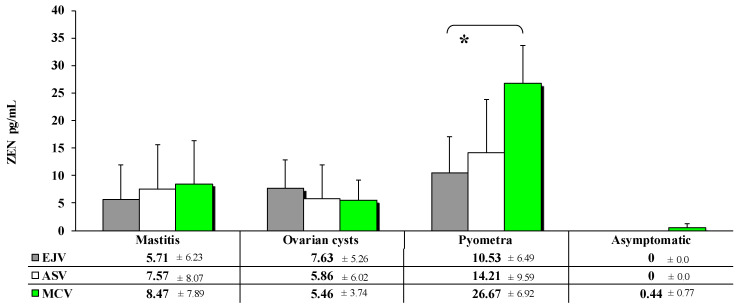
Mean values ($\bar{x}$) and standard deviation (SD) of ZEN concentrations (pg/mL) in peripheral blood of cows collected in various disease states (Mastitis; Ovarian cysts; Pyometra; Asymptomatic) at different collection sites [external jugular vein (EJV-*v. jugularis externa*-posthepatic); abdominal subcutaneous vein (ASV-*v. epigastrica cranialis superficialis*-prehepatic); oraz median caudal vein (MCV-*v. caudalis mediana*-prehepatic)]. Limits of detection (LOD) > values below the limit of detection were regarded as equal to 0. Statistically significant difference was determined at * *p* ≤ 0.05.

A comparison of ZEN concentrations in samples of peripheral blood (Figure 3) collected from diseased cows revealed significant differences (*p* ≤ 0.05) only in the blood of cows with pyometra. The difference between the samples collected from the EJV and the MCV was determined at 16.16 pg/mL. In cows diagnosed with mastitis and ovarian cysts, ZEN concentrations were highly similar (no significant differences) and very low relative to those noted in cows with pyometra.

**Figure 4:**
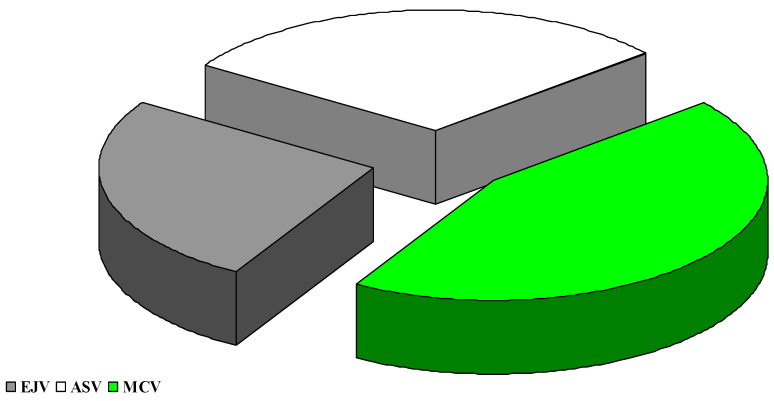
Percentage share of average ZEN concentrations in blood samples collected from different sites (*external jugular vein*-EJV-posthepatic; *abdominal subcutaneous vein*-ASV-prehepatic; *median caudal vein*-MCV-prehepatic) in the monitored herd of dairy cows.

The data presented in Figure 3; Figure 4 show that the highest values of the ZEN level were recorded in Pyometra cows, where the percentage share of the mean values of ZEN concentrations was 44.08%.

The share of individual blood sampling sites in the values of the ZEN level indicates an upward trend, starting with *external jugular vein* (10.53 ng/mL, which constituted 44.13% in this collection site), through the obtained values in *abdominal subcutaneous vein* (14.20 ng/mL, which was 51.39% at this point of samples), and ending with the concentration values obtained in blood samples collected in *median caudal vein* (26.67 ng/mL, which constituted 65.68% at this point of collection).

## 3. Discussion

It is generally believed that ruminants are less susceptible to the harmful effects of mycotoxins than monogastric animals because some mycotoxins are degraded by ruminal microbiota [23]. Despite the above, mycotoxins can induce subclinical conditions in dairy cows [24].

The results of the present study should be interpreted in view of the following observations: (i) the feed administered to dairy cows was probably contaminated with very low doses of ZEN [5]; (ii) this is the first study of the type; therefore, the present findings cannot be compared with published data and have to be interpreted by extrapolation.

According to Fushimi et al. [25], very low levels of ZEN in feed do not affect reproductive performance, but they affect anti-Müllerian hormone levels in the blood [26,27] which play an important role in folliculogenesis and are most highly expressed in the granulosa cells of preantral follicles, mostly in the antral stage [28,29]. These hormones inhibit follicle stimulating hormone (FSH)-induced growth and development of the remaining primary follicles and the selection of the dominant follicle [30,31,32].

In cows, *β*-ZEL is identified more frequently than *α*-ZEL in the intestinal contents contaminated with low doses of ZEN, which implies that detoxification processes are predominant during exposure to very low ZEN doses. It should also be noted that: (i) ZEN could be utilized as a substrate that regulates (inversely) the expression of genes encoding HSDs (Hydroxysteroid Dehydrogenases) which act of molecular switches for the modulation of steroid hormone prereceptors [33,34,35]; (ii) enterohepatic recirculation occurs before ZEN and its metabolites are biotransformed and eliminated; and (iii) in vitro studies have demonstrated that ZEN metabolites are detected within 15 min to 1 h after ingestion, which indicates that this mycotoxin is rapidly metabolized by ruminal microbiota [5,23]. In the present study, the latter observation was confirmed in asymptomatic cows.

These hypotheses (alone or in combination) could explain the differences in the concentrations of ZEN and its metabolites in the peripheral blood of dairy cows exposed to low doses of the mycotoxin. However, these differences could be also attributed to unknown factors that cause rumen inflammations, thus increasing the risk of absorption of toxic compounds, such as lipopolysaccharides (major risk factors) and/or mycotoxins, through the rumen wall [23]. According to Dänicke et al. [4,36], low ruminal pH (which compromises the buffering capacity of the rumen, [23]) inhibits the biotransformation of parent compounds such as ZEN and metabolite synthesis, which could explain the absence of ZEN metabolites in the examined blood samples (Figure 1 and Figure 2). The cited authors also argued that in ruminants, ZEN (parent compound) can be transported to the postruminal digestive tract at unchanged levels and cause mycotoxicosis [37] or digestive disorders contributing to feed-borne diseases such as mastitis, ovarian cysts and pyometra [3]. The above could be explained by a higher rumen passage rate which decreases ruminal pH, thus decreasing the proportion of Gram-positive bacteria and increasing the population of Gram-negative bacteria. These processes occur mainly during the transition period which is accompanied by vast changes in the metabolic processes of cows due to energy, mineral and vitamin deficiencies.

In the present study, ZEN and its metabolites were not detected in asymptomatic cows (Figure 1 and Figure 3), which could be attributed to the rapid biotransformation of mycotoxins by ruminal microbiota or very low concentrations of ZEN in unspecified feed transmission vectors [6]. Similar results were reported in vitro by Debevere et al. [23] who observed that the biotransformation of ZEN to *α*-ZEL and *β*-ZEL (only in the rumen) is highly limited at normal ruminal pH.

Zearalenone metabolites were not detected in diseased cows (Figure 1 and Figure 3). However, trace amounts of the parent compound were identified in the blood, probably because ZEN is not completely metabolized in the rumen. This mycotoxin is transported to successive intestinal segments in unmodified form, and it reaches peripheral organs with the blood and enters the prehepatic circulation that ends in the portal vein (*vena portae*) which supplies blood to the liver [4,22]. The evaluated herd was probably exposed to ZEN for a long period of time or continuously. In laboratory analyses, ZEN is very often detected in maize silage, green fodder and hay [6,37], which indicates that this mycotoxin is present in feed throughout the year. The present results point to dysfunctions of ruminal microbiota or the liver, a unique immunological site that protects the body against mycotoxins. These defense mechanisms can participate in the development of tolerance [38] or initiate a separate immune response [4].

Subclinical disease states caused by mycotoxins should be analyzed in greater detail in high-yielding dairy cows. Disease states can be also caused by saprophytic or conditionally pathogenic microorganisms (bacterial lipopolysaccharides) which contribute to comorbidities, including mycotoxicosis [23]. The etiological factors of disease include an increase in the rumen passage rate caused by increased concentrate intake or the postnatal period (as a result of the physiological loss of the fetus), as well as metabolic disorders such as subacute rumen acidosis resulting from changes (“shift”) in ruminal microbiota [39]. Mycotoxins are less effectively detoxified in the rumen, and they are transported to the intestinal lumen [5]. Zearalenone reaches the intestines and unpaired organs in the abdominal cavity, and it is carried by the prehepatic circulation to the hepatic portal vein and the liver. As a result, substances absorbed from the digestive tract can be more accurately controlled [22].

The above observations were confirmed by ZEN levels in blood samples collected from the MCV of cows with pyometra. These samples were characterized by the highest concentrations of ZEN (Figure 2, Figure 3 and Figure 4). After ingestion, ZEN reaches the proximal and, subsequently, distal segments of the intestines, and it is transported to the liver by prehepatic vessels where it detoxifies from undesirable or dangerous substances as part of the functional circulation. The described hypothesis was also validated by the percentage share of average ZEN concentrations in blood samples collected from diseased cows. In blood sampled from cows with pyometra, the above parameter was determined at 57% (Figure 2), whereas in the remaining (posthepatic) sampling sites, ZEN concentrations (Figure 1 and Figure 3) and their percentage share (Figure 2 and Figure 4) were much lower because the analyzed mycotoxin had already been detoxified in the liver.

## 4. Materials and Methods

All experimental procedures were consistent with Polish regulations defining the conditions and methods of animal experimentation (opinion No. 01/2010/D issued on 21 December, 2016-by the Local Ethics Committee for Animal Experimentation of the University of Warmia and Mazury in Olsztyn, Poland).

### 4.1. Experimental Animals and Feed

The animals were kept in a barn with access to pasture. Blood for toxicological analyses was sampled from cows clinically diagnosed with mastitis (9 animals), ovarian cysts (6 animals), pyometra (5 animals) and from 9 asymptomatic cows (with no clinical symptoms).

The research lasted one year, and it involved 150 dairy cows that were free of clinical symptoms of ZEN mycotoxicosis. The milk yield (herd average) at the end of the last farming year was 9700 L per lactation 305 days (there was an upward trend). All cows are fed the fodder at the bunk feeding based on the average yield of the production group.

There are 3 production groups in the herd: (1)—the most efficient (approximately the first 150 days of lactation); (2)—from day 151 to the end of lactation; (3)—dry cows. The tested dairy cows health problems occurred only in Group 1. Groups 1 and 2 receive an additional amount of total mixed ration (TMR) at the feeding station, depending on their individual performance. Group 3 only receives feed at the bunk feeding. Total mixed ration for dairy cows for all animals in the barn was supplied by the same producer. TMR was administered twice a day, at 6:00 a.m. and 5:00 p.m., in a powdery form. The TMR composition declared by the manufacturer is presented in Table 1.

In addition to the presented TMR composition, the manufacturer also declared the share of components in TMRs for dairy cows, as shown in Table 2.

The approximate chemical composition of the silage to dairy cows (Table 3) was determined using the NIRS™ DS2500 F Feed Analyzer (FOSS, Hillerød, Denmark) which is a monochromatic NIR reflectance and transflectance analyzer with scanning range of 850–2500 nm.

### 4.2. Blood Sampling

In each cow, blood was sampled from three sites in the hepatic portal system [22]: (1) external jugular vein (EJV, *v. jugularis externa*-posthepatic); (2) abdominal subcutaneous vein (ASV, *v. epigastrica cranialis superficialis*-prehepatic); (3) median caudal vein (MCV, *v. caudalis mediana*-prehepatic). Blood samples of 10 mL each were collected from each site into vials containing 0.5 mL of heparin solution. The samples were centrifuged at 3000 rpm for 20 min at a temperature of 4 °C. The separated plasma was stored at −18 °C until sample analysis for the presence of ZEN (according to the schedule of the monitoring program).

### 4.3. Extraction Recovery

The standard addition (fortification) method was used to evaluate the recovery in this study. Non-contaminated sample matrix (blood serum) were enriched by three mycotoxins (ZEN, α-ZEL, β-ZEL) at low (5 pg/mL) medium (10 pg/mL) and high (20 pg/mL) concentrations and pretreated using the methodology outlined in Section 4.4. After analysis, the extraction recovery (ER) method was calculated as: ER = A/B × 100; where A is the slope of the fortified sample after extraction and B is the slope of the fortified sample before extraction. Recovery after extraction from fortified samples ranged from 90% to 102%, suggesting that the pretreatment method met the requirement for mycotoxin determination.

### 4.4. Mycotoxin Extraction

Zearalenone, α-ZEL and β-ZEL were extracted from the blood plasma with the use of immunoaffinity columns (Zearala-TestTM Zearalenone Testing System, G1012, VICAM, Watertown, MA, USA). All extraction procedures were conducted in accordance with the manufacturers’ instructions. The eluates were placed in a water bath with a temperature of 50 °C, and the solvent was evaporated in a stream of nitrogen. Dry residues were combined with 0.5 mL of 99.8% methanol to dissolve the mycotoxins. The procedure were monitored with the use of external standards (Cayman Chemical 1180 East Ellsworth Road Ann Arbor, Michigan 48108 USA, ZEN-catalog number 11353; Batch 0593470-1; *α*-ZEN-catalog number 16549; Batch 0585633-2; *β*-ZEN-catalog number 19460; Batch 0604066-7), and the results were validated by mass spectrometry.

### 4.5. Chromatographic Analysis of ZEN and Its Metabolites

The concentrations of ZEN and its metabolites, α-ZEL and β-ZEL, were determined by the Institute of Dairy Industry Innovation in Mrągowo. Zearalenone and its metabolites were quantified in the blood plasma with the use of combined separation methods involving immunoaffinity columns (Zearala-TestTM Zearalenone Testing System, G1012, VICAM, Watertown, MA, USA), Agilent 1260 liquid chromatography (LC) system, and a mass spectrometry (MS, Agilent 6470) system. The prepared samples will be analyzed with the use of the Zorbax rapid resolution chromatographic column (2.1 × 50 mm; 1.8 micron Agilent Eclipse Plus C18) in gradient mode. The mobile phase will contain 0.1% (*v*/*v*) formic acid in water (solvent A) and 0.1% (*v*/*v*) formic acid in acetonitrile (solvent B). Gradient conditions will be as follows: initially, 20% B that increases to 100% B in 4.0 min and back to 20% B in 0.1 min.

Mycotoxin concentrations were determined with an external standard and were expressed in ppt (pg/mL). Matrix-matched calibration standards were applied in the quantification process to eliminate matrix effects that can decrease sensitivity. Calibration standards were dissolved in matrix samples based on the procedure that was used to prepare the remaining samples. A signal-to-noise ratio of 3:1 will be used to estimate the limits of detection (LOD) for ZEN, α-ZEL and β-ZEL. The LOQ will be estimated as the triple LOD value.

### 4.6. Mass Spectrometric Conditions

The mass spectrometer was operate with ESI in the negative ion mode. The MS/MS parameters were opimized for each compoud. The linearity was tested by a calibration curve including six levels. Table 4 shows the optimized analysis conditions for the mycotoxins tested.

### 4.7. Statistical Analysis

Statistical analyses were performed by the Department of Discrete Mathematics and Theoretical Computer Science, Faculty of Mathematics and Computer Science of the University of Warmia and Mazury in Olsztyn. Plasma concentrations of ZEN and its metabolites were determined in asymptomatic cows and in three groups of experimental cows: (1) with clinically diagnosed mastitis, (2) with clinically diagnosed ovarian cysts, and (3) with clinically diagnosed pyometra. In each cow, blood was sampled from three different sites. The results were expressed as means ($\bar{x}$) with standard deviation (SD). In both cases, the differences between mean values were determined by one-way ANOVA. If significant differences were noted between groups, the differences between paired means were determined in Tukey’s multiple comparison test. If all values were below LOD (mean and variance equal zero) in any group, the values in the remaining groups were analyzed by one-way ANOVA (if the number of the remaining groups was higher than two), and the means in these groups were compared against zero by Student’s t-test. Differences between groups were determined by Student’s t-test. The results were regarded as highly significant at *p* < 0.01 (**) and as significant at 0.01 < *p* < 0.05 (*). Data were processed statistically in Statistica v.13 (TIBCO Software Inc., Silicon Valley, CA, USA, 2017).

## 5. Conclusions

The results of this study suggest that blood samples for toxicological analyses should be collected from the MCV (prehepatic vessel) of clinically healthy cows and/or cows displaying subclinical symptoms of disease (ZEN mycotoxicosis). Blood sampled from the MCV improves the reliability of diagnosis of subclinical ZEN mycotoxicosis.

## Figures and Tables

**Table 1:** Declared total mixed ration for caws.

The Feed Materials Used	
Maize, rapeseed extraction meal, soybean meal, wheat bran, triticale, distillation dried cereal and corn, dried and molasses beet pulp, sunflower meal, wheat mix, beetroot molasses, decoction of sugar beet molasses, rumen-protected fatty acid salts of plant origin, calcium carbonate, sodium chloride and niacin.	
**Ingredients**	**Composition ^1^ Declared by the Manufacturer (%)**
**Barley middling’s**	36.5
**Triticale middling’s**	18.5
**Cornmeal**	18.0
**Post-extraction rapeseed meal**	7.0
**Post-extraction soybean meal**	9.0
**Protein concentrate R-056**	9.0
**Vitamin-mineral supplements ^1^**	2.0

^1^ Composition of the vitamin-mineral supplements per kg: vitamin A—17,500.00 IU; vitamin D3—5000.00 IU; vitamin E (alpha-tocopherol)—100 mg; B3 (niacin)—400 mg; biotin—400.00 µg; iron (iron sulfate) —110 mg; manganese (manganese sulfate)—125 mg; zinc (zinc sulfate)—125 mg; copper (CuSO_4_·5H_2_O)—20 mg; vitamin iodine (potassium iodide)—1.8 mg; selenium (sodium selenate)—0.35 mg; Seldox antioxidatum (BHA-E320, BHT-E321, Ethoxyquin E324)—0.95 mg; flavoring substances—0.5 g.

**Table 2:** Declared total analytical components in total mixed ration for caws.

Components	Analytical Components–Manufacturer’s Declared Composition (%)
**Crude protein**	19.00
**Crude fiber**	6.50
**Raw oils and fats**	4.10
**Crude ash**	6.20
**Sugar**	7.50
**Total calcium**	0.80
**Total phosphorus**	0.60
**General sodium**	0.30
**Total magnesium**	0.30

**Table 3:** The results of the silage analysis in g/kg dry metter.

Indicators	Haylage	Maize Silage
***Dry matter***	***231.05***	***434.24***
***pH***	***6.19***	***4.52***
**Ammonia fraction**	11.95	10.93
**Crude protein**	198.25	80.17
**Crude fiber**	285.09	180.49
**Ash**	99.12	47.20
**Sugar**	-	11.17
**Starch**	-	302.20
**Neutral Detergent Fiber (NDF)**	504.62	386.37
**Acid Detergent Fiber (ADF)**	310.75	217.58
**Acid Detergent Lignine (ADL)**	10.17	22.52
**Crude fat**	32.50	29.99
**Straw.mat org VOS**	682.64	688.52
**Neutral Detergent Insoluble Crude Protein (NDICP)**	38.19	21.92
**Acid Detergent Insoluble Crude Protein (ADICP)**	9.18	7.53
**Lactic acid**	52.91	55.63
**Volatile Fatty Acids (VFA)**	248.88	133.69
**Acetic acid**	18.30	14.76
**Butyric acid**	3.05	-

**Table 4:** Optimized conditions for mycotoxins tested.

Analyte	Precursor	Quantification Ion	Confirmation Ion	LOD (ng mL^−1^)	LOQ (ng mL^−1^)	Linearity (%R^2^)
**ZEN**	317.1	273.3	187.1	0.03	0.1	0.999
**α-ZEL**	319.2	275.2	160.1	0.3	0.9	0.997
**β-ZEL**	319.2	275.2	160.1	0.3	1	0.993

## Data Availability

Not applicable.
